# Identification of 3-((1-(Benzyl(2-hydroxy-2-phenylethyl)amino)-1-oxo-3-phenylpropan-2-yl)carbamoyl)pyrazine-2-carboxylic Acid as a Potential Inhibitor of Non-Nucleosidase Reverse Transcriptase Inhibitors through InSilico Ligand- and Structure-Based Approaches

**DOI:** 10.3390/molecules26175262

**Published:** 2021-08-30

**Authors:** Deepti Mathpal, Tahani M. Almeleebia, Kholoud M. Alshahrani, Mohammad Y. Alshahrani, Irfan Ahmad, Mohammed Asiri, Mehnaz Kamal, Talha Jawaid, Swayam Prakash Srivastava, Mohd Saeed, Vishal M. Balaramnavar

**Affiliations:** 1 School of Pharmacy and Research, Sanskriti University, 28 K. M. Stone, Mathura Delhi Highway, Chhata, Mathura 281401, Uttar Pradesh, India; divyamathpal10@gmail.com; 2 Department of Clinical Pharmacy, College of Pharmacy, King Khalid University, P.O. Box 61413, Abha 62529, Saudi Arabia; talmelby@kku.edu.sa; 3 College of Medicine, King Khalid University, P.O. Box 61413, Abha 62529, Saudi Arabia; Kholoudalbjadi@gmail.com; 4 Department of Clinical Laboratory Science, College of Applied Medical Sciences, King Khalid University, P.O. Box 61413, Abha 62529, Saudi Arabia; moyahya@kku.edu.sa (M.Y.A.); irfancsmmu@gmail.com (I.A.); masiri@kku.edu.sa (M.A.); 5 Department of Pharmaceutical Chemistry, College of Pharmacy, Prince Sattam bin Abdulaziz University, P.O. Box 173, Al Kharj 11942, Saudi Arabia; mailtomehnaz@gmail.com; 6 Department of Pharmacology, College of Medicine, Al Imam Mohammad ibn Saud Islamic University (IMSIU), Othman ibn Affan Street, Riyadh 13317, Saudi Arabia; talhajawaid78@gmail.com; 7 Department of Pediatrics, Yale University School of Medicine CT, New Haven, CT 06520, USA; swayam.cdri@gmail.com; 8 Vascular Biology and Therapeutic Program, Yale University School of Medicine CT, New Haven, CT 06511, USA; 9 Department of Biology College of Sciences, University of Hail, P.O. Box 2440, Hail 55425, Saudi Arabia

**Keywords:** reverse transcriptase, pharmacophore, virtual screening, ligand mapping

## Abstract

Non-nucleosidase reverse transcriptase inhibitors (NNRTIs) are highly promising agents for use in highly effective antiretroviral therapy. We implemented a rational approach for the identification of promising NNRTIs based on the validated ligand- and structure-based approaches. In view of our state-of-the-art techniques in drug design and discovery utilizing multiple modeling approaches, we report here, for the first time, quantitative pharmacophore modeling (HypoGen), docking, and in-house database screening approaches in the identification of potential NNRTIs. The validated pharmacophore model with three hydrophobic groups, one aromatic ring group, and a hydrogen-bond acceptor explains the interactions at the active site by the inhibitors. The model was implemented in pharmacophore-based virtual screening (in-house and commercially available databases) and molecular docking for prioritizing the potential compounds as NNRTI. The identified leads are in good corroboration with binding affinities and interactions as compared to standard ligands. The model can be utilized for designing and identifying the potential leads in the area of NNRTIs.

## 1. Introduction

Reverse transcriptase (RT) inhibitors are among the main targets in contemporary drug discovery efforts against HIV infection. The RT is an important target for viral replication [1]. In the present scenario, where no effective vaccine against HIV is available, the RT inhibitors are up-and-coming agents for use in highly effective antiretroviral therapy (HAART), which is typically a combination of three or four antiretroviral drugs [2,3,4,5,6]. The HAART has significantly reduced the morbidity and mortality of HIV-infected people [7]. Two categories of RT inhibitors are currently in use, including non-nucleoside reverse transcriptase inhibitors (NNRTIs, e.g., Efavirenz, Nevirapine, ά-anilinophenylacetamide-APA, Delavirdine) [8] and nucleosidase reverse transcriptase inhibitors (NRTIs, e.g., Zidovudine, Didanosine, Zalcitabine). The NNRTIs act by binding to an allosteric binding site located 10Ǻ away from the polymerase active site in the NNRTI Binding Pocket (NNRTIBP) [9,10]. The USFDAhave also approved three NNRTIs for HIV-RT for sale in the United States: Nevirapine [11], Delavirdine (a BHAP derivative, BHAP U-90152) [12,13,14], and Efavirenz [15],while some other promising NNRTIs have also been developed, including DABO derivatives [16,17,18,19,20], HEPT derivatives [18,21,22], TIBO derivatives [23], TSAO derivatives [24,25], oxathiincarboxanilide derivatives [26,27,28], quinoxaline derivatives [4,29], thiadiazole derivatives [30], and PETT derivatives [31].

In recent years, drug discovery approaches, including advanced computer-aided drug design-guided structure–activity relationship studies, have facilitated many NCE drug discoveries and innovation in the diverse classes of diseases, such as Alzheimer’s disease (AD), cancer, anddiabetes [32,33,34,35,36]. The ligand-based drug design can possibly be performedwith the software programs such asSYBYL/comparative molecular field analysis (CoMFA) [37], comparative molecular similarity indices analysis (CoMSIA) [38], and Catalyst/HypoGen (quantitative) and HipHop (qualitative) [39]. Both of these techniques have suffered from few limitations. Pharmacophore query-based virtual screening methods are well-documented, accepted, and found superior in their screening ability on extensive databases, being faster and able toretrieve more structurally diverse leads than structure-based methods, CoMFA, and CoMSIA [40,41,42].

The reverse transcriptase enzyme is the significant target for pharmacophore design as NNRTIBP is known to be flexible and tomoveto accommodate inhibitors, acquiring different shapes depending on the bound inhibitor [43]. Therefore, in such a condition, a ligand-based drug design approach where the 3D structural features of ligands are considered to develop pharmacophores, which may provide vital information for the design of new ligands, can be developed. The non-nucleoside binding site (NNBS) may be considered a rigid pocket in developing such a pharmacophore. A 3D arrangement of chemical features in the molecules is essential for important binding interactions with the RT enzyme. Based onthe above and considering the limitations of structure- and ligand-based approaches, we have devised a hybrid approach utilizing both approaches’ mutual strengths, simultaneously compensating for their limitations. Due to their high antiviral potency, in previously published work, we carried out HypoGen pharmacophore modeling of 4-Benzoyl-3-dimethylamino pyridine-2 (1H) [22] as a potent reverse transcriptase inhibitor, followed by its implementation in virtual screening with the focused library as well as commercial databases, which were finally validated by structure-based modeling using known protein structures both for the wild-type HIV-RT and mutant PDBs. The pharmacophore-based virtual screening (PBVS) and the docking-based virtual screening (DBVS) have been recently implemented and reported by our group to discover novel PTP1B, AchE, and Caspase-3 inhibitors for diabetes and Alzheimer’s disease [44,45,46]. The state-of-the-art techniques in scaffold hopping, focused library design and its synthesis, followed by HIV-RT inhibitory activity, are reported in this work.

## 2. Methodology

### 2.1. Computational Details

#### 2.1.1. Data Selection

The most critical aspect in the generation of the pharmacophore hypothesis using the Catalyst program is selecting the training set. Some basic guidelines have been suggested for the selection of the training set, e.g., a minimum of 16 diverse compounds to avoid any chance correlation, the activity data should have a range of 4–5 orders of magnitude, the compounds should be selected to provide clear and concise information to avoid redundancy or bias in terms of both structural features and activity range, and most of the highly active compounds should be included so that they provide information on the most critical features required for a reliable/rational pharmacophore model [24]. In view of the above, the series of NNRTIs reported by Benjahad et al. [23] consisting of 103 compounds were chosen for the present study. It is important to note that some compounds in this series are even more potent than the known drugs (Efavirenz and TIBO, etc.).

#### 2.1.2. Generation of Pharmacophore Hypothesis

The structures of all the compounds were built and geometry-optimized using Catalyst 4.11. All the compounds were minimized to the closest local minimum using the Charm-M-like force field incorporated in the Catalyst program. As a prerequisite to the hypothesis generation by Catalyst, diverse conformations were generated for the compounds (255 for each) using the poling algorithm [47] (BEST) to cover the conformational space within the energy threshold of 20.0 Kcal/mol above the global energy minimum. This method will penalize any newly generated conformer which is too close to any already found conformers. This method ensures maximum coverage in conformational space. All other parameters were set to the default settings. The conformations generated by using the procedure described above were used for the hypothesis generation using the default uncertainty value of 3. Before generating the quantitative model for HIV-RT inhibitors, the common feature hypothesis was carried outto identify the requisite features for anti-reverse transcriptase activity. The best HIP-HOP model generated (not provided here) contains five types of chemical features, namely, hydrogen-bond donor (D), hydrogen-bond acceptor (A), two hydrophobic aliphatic (Z), and ring aromatic (R) features. Based on this information, the initial quantitative hypothesis suggested thathydrogen-bond acceptor lipid (HBAL), hydrophobic (HY), and aromatic ring features can map essential features of all of the compounds in the dataset. These features were used to generate 10 predictive hypotheses (HypoGen) using the training set compounds. The minimum and maximum count of features for HY were0 and 5 respectively, whereas for HBAL, the values were 0 and 3, respectively. Pharmacophore generation was carried out by using the default parameters and the setting implemented in the HypoGen generation procedure of the Catalyst program, except for the inter-feature distance, where a default value (2.97 Å) was reduced to 2 Å due to the small molecular size of the active compounds used in the training set. The choice and number of features used in the hypothesis construction were hydrogen-bond acceptor lipid (HBAL), hydrophobic (HY), and ring aromatic. A default activity uncertainty value of 3 has been used in the pharmacophore generation. The specifications regarding the pharmacophore generation have been well-documented by Kristam et al. to perform the reproducibility of the pharmacophore [32].

#### 2.1.3. Cat Scramble Validation (Fisher Test)

The model was evaluated for statistical relevance by Fisher’s randomization test. This test involves thorough randomization of the training set to validate and derive the significance of the generated best model. Consequently, the pharmacophore model corresponding to the Hypo-1 was evaluated for statistical significance using a randomization trial procedure derived from the Fisher method [24]. These randomized spreadsheets should yield hypotheses with lesser statistical significance than the original model to suggest that the original hypothesis represents a true correlation. The number of such random trials depends on what level of statistical significance is to be achieved. For a 95% confidence level, 19 spreadsheets are created, while for 98% and 99% confidence levels, 49 and 99 spreadsheets are created, respectively. Our model was found to be 99% significant in the F-randomization test, which substantiates the significance of the model.

### 2.2. Molecular Modeling and Docking Studies

MolDock is a docking module of Molegro Virtual Docker (MVD) software (Thomsen et al., 2006). It is based on a new hybrid search algorithm, called guided differential evolution (DE). The guided DE algorithm combines the DE optimization techniques with a cavity prediction algorithm. DEwas introduced by Storn and Price in 1995 and has previously been successfully applied to molecular docking [33]. The use of predicted cavities during the search process allows for fast and accurate identification of potential binding modes (poses). The docking scoring function of MolDock is based on a piecewise linear potential (PLP) introduced by Gehlhaar et al. [34,35]. In MolDock, the docking scoring function is extended with a new term, taking hydrogen-bond directionality into account.

Moreover, a re-ranking procedure was applied to the highest-ranked poses to further increase docking accuracy. The reported crystal structures of 1JKH and 1DTQ were obtained from Brookhaven Protein Data Bank (PDB). Initially, the protein was considered without ligand and water molecules. The backbone was fixed, the Charm-M force field and minimization using a steep descent algorithm were applied for protein structures, and all the inhibitor structures were prepared using the Charm-M force fields and minimized up to agradient of 0.01 kcal/(mol Å) with the help of Discovery Studio 2.0 software (Telesis Court, San Diego, CA, USA). Due to the availability of the co-crystallized structure ofHIV-1 reverse transcriptase in complex with DMP-266 andPETT-1, we used the template docking available in the MolegroVirtual Docker and evaluated the MolDock, re-rank, and protein–ligand interaction scores from MolDock(GRID) options. Template docking is based on extracting the chemical properties, such asthe pharmacophore elements, of a ligandbound in the active site. This information is utilized in the docking of structurally similar analogs. TheDMP-266 andPETT-1 models from 1JKH and 1DTQ [4,29] wereused as the template with the default settings, including a grid resolution of 0.30, for grid generation, and a11 Å radius from the template as the binding site. MolDock SEwas used as a search algorithm, and the number of runs was set to 10. Apopulation size of 50 and a maximum iteration of 1500 were used for parameter settings. The maximum number of poses generated was 10. Since theMolegroVirtual Docker works by an evolutionary algorithm, consecutive docking runs do not yieldthe same poses and interactions. To address this inherent randomness, three consecutive runs were performed, and the top three poses were used to visualize the interactions of HIV-RT inhibitors.

## 3. Results

### Pharmacophore Generation

**Figure 1:**
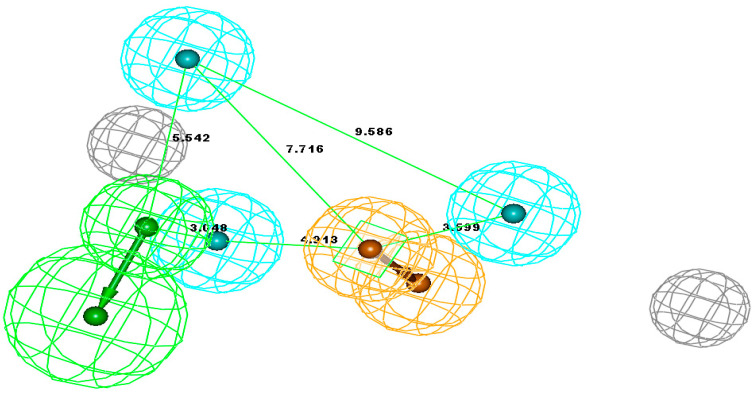
The best hypothesis = Hypo-1. Pharmacophore features: Green—HBAL, Orange—RA, blue—HY. Distance between pharmacophore features is indicated in angstrom (Ǻ).

The pharmacophore studies were performed using the series of NNRTIs reported by Benjahad et al. [23], consisting of 103 compounds, which resulted in the critical features required for a reliable/rational pharmacophore model (Table 1). The biological activity data spanning over 5 orders of magnitude (0.0004–100 μM) and various molecular features make this dataset highly suitable for the development of predictive pharmacophore model(s) with the Catalyst HypoGen algorithm. The training set (30 compounds) was selected considering the above guidelines, while the rest of the compounds were kept aside as a test set (73 compounds) for the validation of the pharmacophore models (Table 1). The initially generated hypothesis suggested thathydrogen-bond acceptor lipid (HBAL), hydrophobic (HY), and ring aromatic (RA) (Figure 1) were able to map important features of all of the compounds in the dataset. These features were used to generate 10 predictive hypotheses (HypoGen) using the training set compounds. The null, fixed, and configuration costs were found to be 203.238, 115.403, and 13.075, respectively. The total cost ranged from 146.254 to 180.362 for the 10 hypotheses. In comparison, the difference between total and null cost was found to be >40 for the first 7 hypotheses out of the 10 generated, indicating that these hypotheses (first 7) have at least a 75–90% probability of representing true correlation in the data (Table 2). The lowest RMS deviation and the best correlation coefficient were found to be 1.43 and 0.836, respectively. The cost values, correlation coefficients, and different pharmacophoric features for generating the hypothesis are reported in Table 2.

The first seven hypotheses which have a cost difference of >40 can be further classified into two distinct groups, i.e., group one consisting of hypotheses having HBAL, HY, HY, HY, and RA features (hypotheses 1, 2, 4, and 5), while the second group is characterized by HBD, HY, HY, HY, and RA features (hypotheses 3, 6, and 7). The first 5 hypotheses have the best overall results in terms of cost difference (>50) and higher correlation coefficient (>0.80). The first hypothesis showed the cost difference (Δ = 55.084), correlation coefficient (r = 0.84), and consists of five features, including one HBA-lipid, three hydrophobic, and a ring aromatic feature (Figure 1). Since this hypothesis has the highest cost difference, therefore, it was taken as a representative hypothesis for the first group; similarly, hypothesis 3 was chosen as a representative hypothesis for the second group. Hypothesis 1 was found to rank the compounds in a better manner and hence it is discussed in detail below (Supplementary Table S1).

To test the ranking efficiency of the hypotheses, the compounds of the training and test sets were classified as HA (highly active, +++, 0.0004–0.01), MA (moderately active, ++, 0.01–5), and LA (least active, +, 5–100) according to their reported biological activity (Supplementary Table S2). The training set compounds along with their fit values and mappings to the pharmacophore are providedin Supplementary Table S1. Hypothesis 1 was found to rank all the compounds of the training set correctly into their respective classes (HA, MA, and LA).

## 4. Discussion

**Figure 2:**
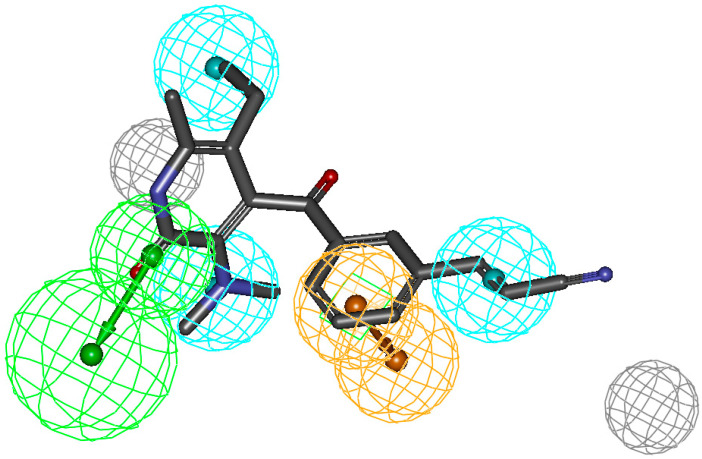
Pharmacophore mapping of the most active (**27**) compound from the training set predicted correctly. The figure indicates the mapping of all groups essential for receptor binding. Reported activity: 0.0004 μM, estimated: 0.0009 μM, and fit value: 13.45.

A close examination of the mappings reveals that the compounds of the training set map four functions (HBAL, RA, and HY A, B and C, Figure 2). Therefore, it appears that these four features are essential for anti-RT activity. The LA compounds in the series such as **83** (fit value 6.97) have an imidazole ring in the place of a benzene ring and the dimethyl group on the parent aromatic benzene ring. This imidazole ring cannot map the RA feature of the hypothesis and is not capable of making hydrophobic interactions as strong as the phenyl ring; thus, the compounds withthe imidazole ring generally have a lower fit value than the compounds witha phenyl ring. In the case of **84**, the –CH_3_ group of the imidazole ring is in close proximity to 2-dimethyl groups on tertiary nitrogen, which create steric hindrance due to proximity and thus offer less surface area to access as compared to **27** (Figure 2), which is highly active. The compound **89** in the training set was predicted as HA as the substitution by Br present on the ring system in place of –CH_3_ may provide more hydrophobicity and thus maps the pharmacophore better. Additionally, the presence of bromine assures less steric hindrance with the C=O group and offers additional stability. The most active compound has a fitness score of 13.45 (**27**), while the second-best (**61**) has a fitness of 12.18 and both of the compounds are ranked correctly as HA.

### 4.1. Cat Scramble Validation (Fisher Test)

**Figure 3:**
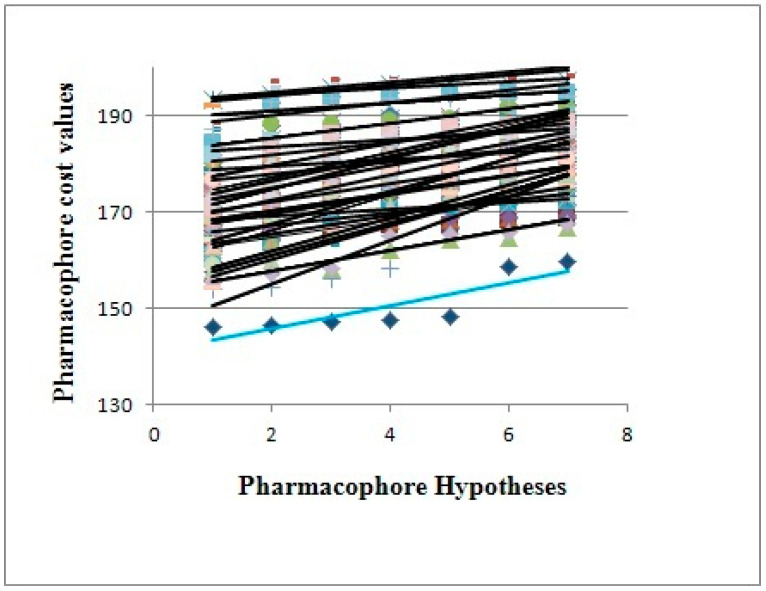
The cost difference between Hypo-1 (sky-blue color) and the scrambled runs (other colors).

The model was evaluated for statistical relevance by Fisher’s randomization test. This test involves thorough randomization of the training set to validate and derive the significance of the generated best model. Consequently, the pharmacophore model corresponding to the Hypo-1 was evaluated for statistical significance using a randomization trial procedure derived from the Fisher method. These randomized spreadsheets should yield hypotheses with lesser statistical significance than the original model to suggest that the original hypothesis represents a true correlation. The number of such random trials depends on what level of statistical significance is to be achieved. For a 95% confidence level, 19 spreadsheets are created, while for 98% and 99% confidence levels, 49 and 99 spreadsheets are created, respectively. Our model was found to be 99% significant in the F-randomization test, which substantiates the significance of the model (Figure 3).

### 4.2. Validation by Test Set

The most critical objective of pharmacophore generation in virtual screening studies is to classify the molecules as active and inactive with high accuracy. Therefore, a large test set (73 compounds) was used to test the predictive power of the generated pharmacophore model. The best pharmacophore was chosen to estimate the activity of the test set. The activity value of test set compounds was estimated using the best fit procedure as implied in Catalyst. Out of 32 highly active compounds, 29 were predicted correctly as HA, while the other 3 were predicted as MA. However, out of 40 moderately active compounds (MA), 28 were predicted as HA while 12 were predicted as MA, and none were predicted as LA (Supplementary Table S2). Therefore, it can be said that the generated pharmacophore model is highly capable of accurately classifying molecules as active or inactive NNRTIs and can be used for virtual screening purposes.

### 4.3. Further Validation and Mapping Studies Using Standard Compounds

Since the model was able to classify most of the compounds correctly in their respective classes, it appeared of interest to test whether it can identify other compounds which are active against HIV-RTase, since this may indicate the true utility of the generated pharmacophore model. In this endeavor, the pharmacophore model was tested against well-known potent HIV-RTase inhibitors such as Efavirenz and HETP, etc., (Supplementary Table S3). The pharmacophore model correctly classified these compounds as active, and none of the molecules wereclassified as inactive, establishing confidence and broad applicability of the generated model. In the case of the Efavirenz, the features HBAL, RA, and hydrophobic map well, as is the case with HETP. This indicates the high 3D similarity along with important inter-feature distances among these compounds (HIV-RTase inhibitors).

**Figure 4:**
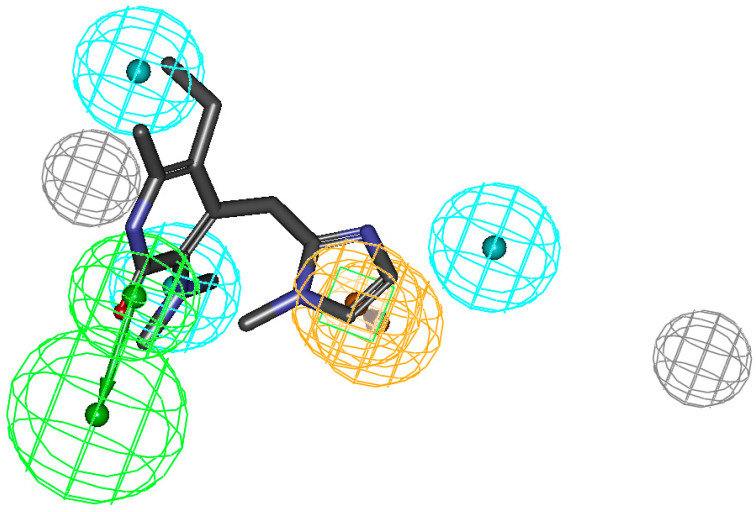
Pharmacophore mapping of the least active compound (**83**) from the training set, predicted correctly. Figure indicates the miss of one hydrophobic group essential for receptor binding. Reported activity: 100 μM, estimated: 36 μM, and fit value: 6.97.

The mapping clearly shows that the bridged side chain needs further modification to map correctly onto the ring aromatic feature, and this information may help in designing compounds with improved activity. The fit values of these compounds to Hypo-1 along with experimentally derived IC_50_ values are providedin Supplementary Table S3. The pharmacophore so developed in this case was found to map all six compounds well, which are known HIV-RTase inhibitors. In the case of the mapping of the LA compound (**83**) from the series, it was found that the molecule lacks some of the features of the model derived frommapping in this way, and wascorrectly predicted asleast active (Figure 4).

### 4.4. Comparison of Generated Pharmacophore Vis-a-Vis Interactions at the Active Site

**Figure 5:**
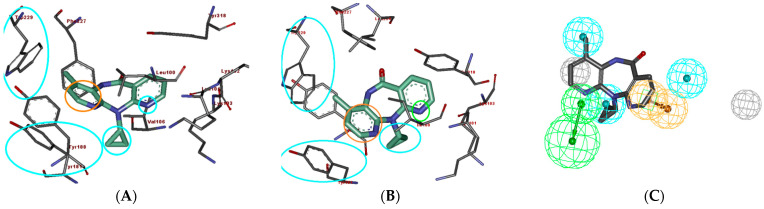
(**A**) PDB ID:1IKW, the co-crystal structure of Nevirapine and theNNRTase active site with important binding interactions [48]. (**B**) The co-crystal structure of Nevirapine and theNNRTase active site with important binding interactions, PDB ID: 1S1U [25]. (**C**) Comparison of the pharmacophore mapping of Nevirapine on Hypo-1.

To study the SAR and the binding patterns, interactions, and pharmacophore mapping of Nevirapine in both wild-type HIV-RT as well as mutant organisms, the molecular docking of Nevirapine in the wild-type HIV-RT PDB ID 1IKW [48] was carried out and compared with the Nevirapine in the X-ray crystal structure (PDB ID: 1S1U) [25], asshown in Figure 5A,B. In both of the structures (wild- and mutant-type), the ring aromatic feature of the hypothesis indicates the interaction between the ligand and the phenyl ring of Tyr181. The two hydrophobic features of the hypothesis, which are mapped on Nevirapine with one at the cyclopropyl ring and the other one at the methyl group, can be seen matching very well at the active site. The cyclopropyl ring of Nevirapine is situated in the vicinity of amino acids Leu100 and Val106 for wild-type and Ile100 and Val189 for the mutant-type, which is hydrophobic in nature; similarly, the methyl group of Nevirapine in both the wild- and mutant-types is surrounded by Trp229 and Tyr188, making good hydrophobic interactions. The nitrogen of the right pyridyl ring is situated close to Lys103, which is a positively charged amino acid with a strong capability of making hydrogen-bonding interactions, thus the HBAL feature is also detected very well on the compound. It is pertinent to note that both of the pyridyl rings of Nevirapine have nitrogen at similar positions, however, only the one which is near Lys103 can make HB interactions, while the other one cannot because of the surrounding hydrophobic residues. This is a very small but subtle difference that was correctly identified by the generated pharmacophore model, which strengthens it. It is to be noted that the pharmacophore was generated using ligand information only; however, it matches the pharmacophoric feature at the HIV-RTase active site very well, which demonstrates the strength of the pharmacophore modeling approach in general.

### 4.5. In-House Database-Based Virtual Screening

**Figure 6:**
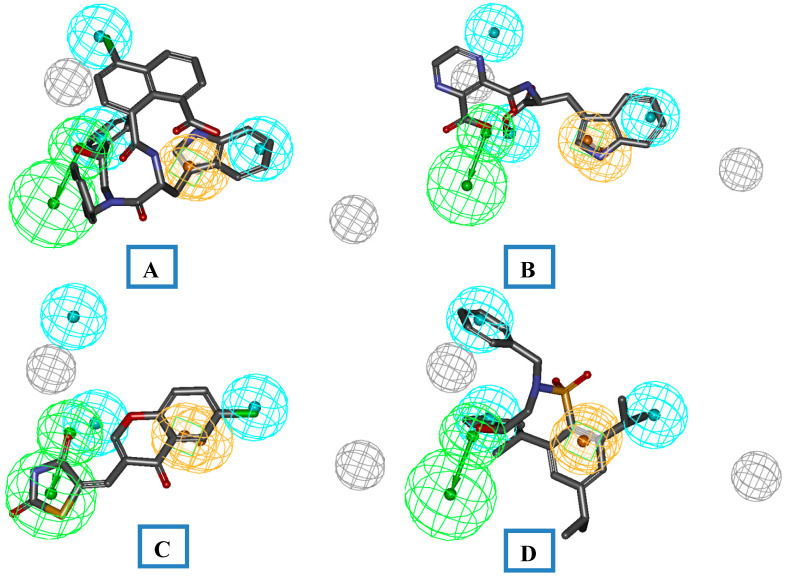
(**A**,**B**) The pharmacophore mapping of each identified core I (Compound **112**, fit value: 9.36382, and Compound **114**, fit value: 8.11322). (**C**) Core II (Compound **128**, fit value: 7.36382). (**D**) Core III (Compound **125**, fit value: 9.12313).

A virtual screening approach is an important approach to the identification of leads. We employed this validated pharmacophore model in the screening of an in-house database of compounds to identify and prioritize the probable lead candidates as HIV-RT inhibitors. These databases were prepared by using the Charm-M force field and used for searching new leads by employing validated pharmacophoric queries in the ligand pharmacophore mapping protocol, employing the flexible fit method by using default settings. Twelve compounds from three different cores were identified from this screening, andthe identified compounds were further prioritized for synthesis. The synthesized compounds were further screened for invitro HIV-RT activity. The detailed synthetic scheme for the synthesis of identified leads is not the part of this manuscript. The identified cores were synthesized by using the protocols reported by our group. The pharmacophore mapping of these compounds along with the predicted activity is shown in Figure 6. The pharmacophore mapping of these compounds reveals that the most active compounds, **112**, **114**, **115**, and **128**,were capable of mapping all the required features of Hypo-1. The basic aromatic ring of the 4-Chloro-1,8-naphthalic acid part of compound **112** serves the requirement of one hydrophobic function of the pharmacophore model. The one –COOH group of the 1-naphthoic acid part of compound **112** maps the one HBA functionality of the pharmacophore model. The indole ring of the tryptophan moiety maps the RA function of the pharmacophore model. The two aromatic rings of the 2-(benzylamino)-1-phenylethanol compensate for the two HY functions of the pharmacophore model (Figure 6A). The other identified molecules from the above screening also map the pharmacophore model, as represented in Figure 6A–D.

### 4.6. Structure-Based Analysis of the CoresI, II, and III

**Figure 7:**
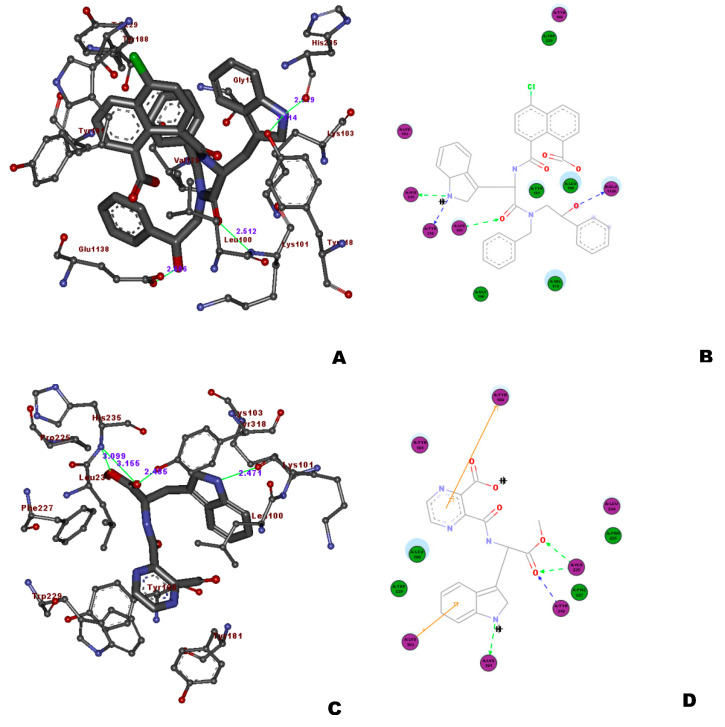

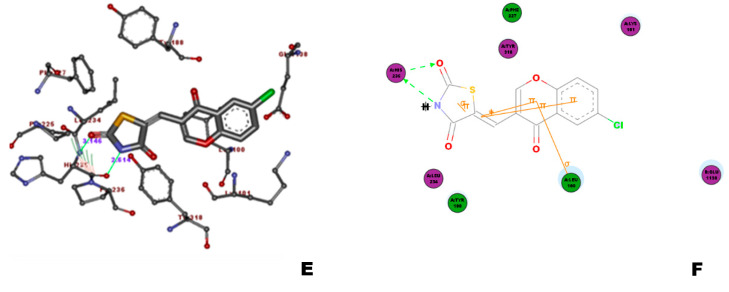
The molecular interactions of the (**A**,**B**) Core I compound **112**, (**C**,**D**) core II compound **114**, and (**E**,**F**) core III compound **128** in the binding site of HIV-RT using the PDB ID: 1IKW.

In continuation of the ligand-based screening to further confirm the binding affinity and the binding interactions of these ligands, the docking studies were carried out. The docking studies were validated using the two PDB IDs (1JKH [4] and 1DTQ [29]) and the template docking protocol of MolegroVirtual Docker 4.0. The docking analysis of three top-ranking compounds from pharmacophore mapping on 1DTQ is discussed in this section. The top-ranked ligand from this study, compound **112**, showed three important hydrogen-bond interactions with the amino acids, Lys103, Lys101, and Lys-100. The 8–COOH group of the napthoic acid showed additional hydrogen-bond interactions with Glu138 and Lys101. The ring aromatic feature of the hypothesis supported by the indole ring of the tryptophan amino acid part of the molecule showed the interaction between the ligand and the phenyl ring of Tyr181, and the –NH group of the indole ring showed the additional hydrogen bond with Tyr188. Similarly, the aromatic ring moieties of thenapthoic acid part along with the –Cl group situated at the 5-position of the compound showed hydrophobic interactions with Val179 andLys101 (Figure 7), while the indole moiety group is surrounded by Trp229 and Tyr188,leading togood hydrophobic interactions. All these interactions were in good correlation with the Nevirapine interactions studied in 1S1U^31^ (Figure 6A). The other core II structure, compound **130**, showed the same interaction pattern with three hydrogen-bond interactions with amino acids, such as Lys101 and Lys-100, along with one additional hydrogen bond with Pro236, while havinggood hydrophobic interactions with Trp229 and Tyr188. The core III compound **129** also showed the interactions with the important amino acid Lys101, along with two additional hydrogen-bond interactions with Tyr319 and Ile135, while the aromatic ring leads tohydrophobic contacts with Tyr319, Pro321, and Lys101.

## 5. Database Screening

The database (Zinc Natural Product and Across database) screening of the pharmacophore query along with template docking gives rise to several unknown compounds which have not yet been documented for anti-HIV activity but were predicted active. The structures of some of the compounds are shown in Table 3. The preliminary filtration by Lipinski’s rule of five resulted in the selection of hits from the NIH and Interbio sciences compound database [30]. The Efavirenz or DMP266 was docked as a reference ligand in the binding site of 1JKH using GOLD and Molegro docking protocols. The GOLD score, MolDock score, and re-rank scores were employed for analysis of the various scores.

**Figure 8:**
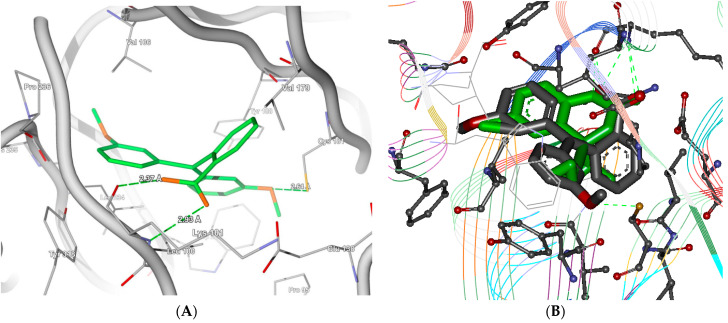
(**A**,**B**). The comparative docking of screened (**A**) ligand (NCI-21270) and (**B**) Nevirapine as a standard drug shows butterfly conformation and important hydrophobic interactions, along with hydrogen-bond interactions with Lys101 and other important residues in the NNRTI’s binding site.

The major interactions of the screened ligands were found with Lys101, Leu100, Thr139, Try383, Val179, Arg172, and Glu28 (Figure 8). Similarly, the leads screened from the dataset were docked in the same binding site using both GOLD and Molegro docking protocols [31,32]. Table 3 enlists the ligands retrieved after the docking along with their GOLD, MolDock, binding affinity, and mapping scores with the pharmacophore model, respectively. The top 49 leads with a GOLD score and MolDock scoreshigher than the reference were identified. All the selected ligands show important binding interactions with Lys101 and Leu103. The docking analysis clearly shows the important interaction of first-generation NNRTIs with an allosteric hydrophobic pocket (non-nucleoside binding site, NNBS) and binging ofthe enzyme in a “butterfly-like” mode. One wing of this butterfly is comprised of an electron-rich (phenyl or allyl substituents) moiety and the other interacts through π–π stacking interactions with a hydrophobic pocket formed mainly by the side chains of aromatic amino acids (Tyr181, Tyr188, Phe227, Trp229, and Tyr318). The top screened leads (Table 3) validated by two docking software programs showed similar interactions with the hydrophobic pocket. The other wing is generally heteroaromatic/aromatic, capable of donating or accepting the hydrogen bonds with Lys101 and Lys103 (Figure 8). The remaining amino acids, such as Lys103, Val106, and Val179, affordadditional hydrophobicity to the butterfly body.

The core of both structures involves butterfly conformation in the binding site, and the hydrophobic and electronegative interactions due to the cyclopropyl ring are well-supported by phenyl ring and carboxylic acid substitution. The better scores in terms of docking for these ligands were due to additional interactions of leads which tend to stabilize binding additions to important core interactions. Thus, these potential leads compriseimportant pharmacophore features required for selective reverse transcriptase inhibition. The comparable docking figures (Figure 8) show the interactions of Nevirapine and the top screened lead with important interactions, and the respective binding scores in terms of GOLD score, MolDock score, and re-rank scores are presented in Table 3.

**Figure 9:**
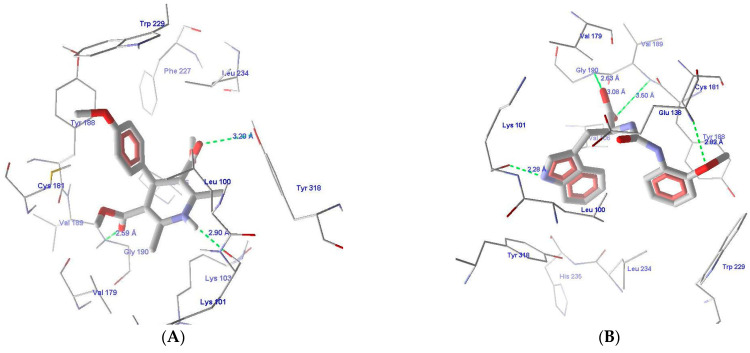
The comparative docking of screened ligands (**A**) ZINC19286543 and (**B**) ZINC02146330 showed butterfly conformation and important hydrophobic interactions, along with hydrogen-bond interactions with Lys101 and other important residues in the NNRTI’s binding site.

The database screening resulted in the identification of 54 compounds as potential NNRTIs. The identified leads were analyzed for their binding interactions with amino acids such as Lys101, Leu100, Thr139, Try383, Val179, Arg172, and Glu28. These ligands were also checked for their affinity in terms of MolDock and re-rank scores. The detailed scores and the top two ligands with their interactions are represented in Figure 9. The two top-scoring compounds from the database screening, ZINC02146330 (MolDockscore:–148.393, re-rank score:−105.048) and ZINC19286543 (MolDockscore:−137.85, re-rank score:−102.759) showed good binding interactions with amino acids such as Lys101, Cys181, Gly190, and Tyr318 (Figure 9A,B). The molecule also showed the stearic and hydrophobic interactions with amino acids, as described in previous sections.

The screening resulted in the identification of the following leads (Table 4) as potential inhibitors of NNRTIs.

## 6. Conclusions

The pharmacophoric model was generated by using 30 diverse training dataset molecules out of 103. The validity of the pharmacophore model was ascertained by: (a) Fisher’s validation, (b) test set prediction, (c) validation by an external dataset of standard molecules, (d) compared validation of pharmacophores with binding site interactions, (e) the in-house database-based screening for identification of the probable hits, (f) design and synthesis and biological evaluation of the identified leads (the data of the invitro studies arenot part of this manuscript), and (e) final applications of the validated pharmacophore model in virtual screening comprised of NCI, Across, Zinc Natural Products, and Inter-Biosciences databases for identification of different NCEs. The well-validated protocol of PBVS resulted in the prioritization of NCEs that tested positive for invitro analysis, and three molecules were analyzed for their selectivity index determination, and compounds **112** and **128** were good RT inhibitors as compared to the marketed drug Nevirapine (the data of the invitro studies arenot part of this manuscript). Atotal of 26 compounds from the Zinc database were curated from a commercial source. This study thus depicted the potential of these compounds to be possible lead compounds and anti-HIV drug candidates.

## Figures and Tables

**Table 1:** Structures of compounds used for pharmacophore model development.

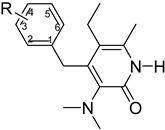							
Structure No.-1-27 Basic Ring				Structure No.-28-59 basic ring			
**Comp.**	**R**	**IC_50_**		**Comp.**	**R**	**IC_50_**	
**1**	3,5-CH_3_	0.008		**28**	3-CH_3_	0.002	
**2**	H	0.004		**29**	3-Br	0.002	
**3**	2-CH_3_	0.025		**30**	3-CN	0.002	
**4**	3-CH_3_	0.002		**31**	4-CN	3.162	
**5**	4-CH_3_	0.316		**32**	3-NO_2_	0.002	
**6**	3-CF_3_	0.01		**33**	3,5-CH_3_	0.004	
**7**	4-CF_3_	1.585		**34**	3,5-Cl	0.002	
**8**	4-C_6_H_5_	3.98		**35**	2,6-F	0.05	
**9**	2-Cl	0.006		**36**	3-F-5-CF_3_	0.003	
**10**	2-Br	0.006		**37**	3-CH_3_-4-OCH_3_	0.079	
**11**	3-F	0.002		**38**	3-N(CH_3_)_2_	0.398	
**12**	3-Cl	0.005		**39**	3-NH_2_	0.012	
**13**	3-Br	0.004		**40**	3-N (C_2_H_5_)	1.995	
**14**	4-Cl	0.199		**41**	3-NHCOCH_3_	0.126	
**15**	4-Br	1.585		**42**	3-NHSO_2_CH_3_	1	
**16**	3-OCH_3_	0.004		**43**	3-NHCONHC_2_H_5_	1.995	
**17**	3-OC_2_H_5_	0.013		**44**	3-(1-pyrrolidinyl-2-one)	0.04	
**18**	4-N(CH_3_)_2_	1.259		**45**	3-(1-pyrrolyl)	0.079	
**19**	2,3-CH_3_	0.1		**46**	3-CH_2_NH_2_	0.398	
**20**	2,5-CH_3_	0.04		**47**	3-CH_2_NHCOCH_3_	0.398	
**21**	3,4-CH_3_	0.04		**48**	3-C_6_H_5_	0.398	
**22**	2,4-CH_3_	10		**49**	3-(2-furyl)	0.063	
**23**	2,4,6-CH_3_	10		**50**	3-(2-thiazolyl)	0.251	
**24**	3,5-F	0.013		**51**	3-(3-pyridyl)	0.006	
**25**	3,5-Cl	0.008		**52**	3-phenylethinyl	0.158	
**26**	3-CH_3_-4-OCH_3_	0.398		**53**	3-CHO	0.008	
**27**	3-CH=CHCN (E)	0.0004		**54**	3-CH_2_OH	0.05	
				**55**	3-COCH_3_	0.01	
				**56**	3-CH_2_CN	0.001	
				**57**	3-CH (CH_3_) CN	0.003	
				**58**	3-CH2OPh	0.039	
				**59**	3-CH (OH) CH_3_	0.05	
Geometric isomers *(continued).* 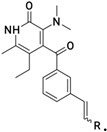							
**Comp.**	**R(E/Z Form)**	**IC_50_**		**Comp.**	**R(E/Z Form)**	**IC_50_**	
**60**	CN (Z)	0.004		**67**	PhCH_2_ (Z)	1.585	
**61**	CN (E)	0.001		**68**	C_6_H_5_ (Z)	1.258	
**62**	-COOEt (E)	0.003		**69**	C_6_H_5_ (E)	0.251	
**63**	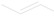 **COOEt**	0.005		**70**	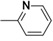 (Z)	1.585	
**64**	H	0.002		**71**	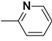 (E)	0.079	
**65**	CH_3_ (E)	0.005		**72**	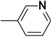 (Z)	0.079	
**66**	CH_3_CH_2_ (E)	0.050					
Geometric isomers *(continued).* 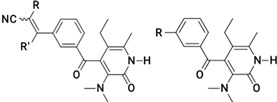							
**Comp.**	**R (E/Z Form)**	**IC_50_**		**Comp.**	**R (E/Z form)**	**IC_50_**	
**73**	CH_3_	0.001		**78**	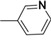 (Z)	0.063	
**74**	CN	0.012		**79**	H (E)	0.001	
**75**	COOC_2_H_5_ (Z)	0.158		**80**	CH_3_	0.006	
**76**	C_6_H_5_ (z)	1		**81**	H	0.003	
**77**	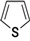 (E)	0.199		**82**	CH_3_	0.008	
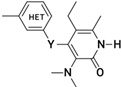							
**Comp.**	**Heterocycle**	**Y**	**IC_50_**	**Comp.**	**Heterocycle**	**Y**	**IC_50_**
**83**	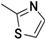	**CH_2_**	0.063	**87**	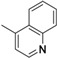	**CH_2_**	5.012
**84**	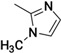	**CH_2_**	100	**88**	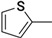	**CO**	0.016
**85**	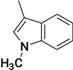	**CH_2_**	0.039	**89**	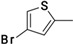	**CO**	0.003
**86**	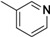	**CH_2_**	0.015	**90**	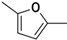	**CO**	0.010
Geometric isomers *(continued).* 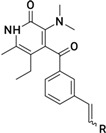							
**Comp.**	**R (E/Z Form)**	**IC_50_**		**Comp.**	**R(E/Z Form)**	**IC_50_**	
**91**	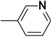 (E)	0.003		**98**	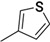 (E)	0.100	
**92**	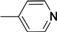 (Z)	0.061		**99**	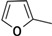 (Z)	0.063	
**93**	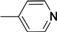 (E)	0.010		**100**	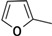 (E)	0.02	
**94**	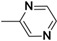 (Z)	0.199		**101**	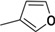 (E)	0.079	
**95**	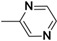 (E)	0.020		**102**	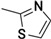 (Z)	0.158	
**96**	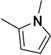 (E)	0.316		**103**	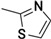 (E)	0.032	
**97**	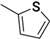 (E)	0.079					

**Table 2:** Results of pharmacophore hypothesis for reverse transcriptase inhibitors.

Hypo	Total Cost	Cost Diff. (Δ) (Null ^a^–Total)	RMS Deviation	Error	Correlation	Features ^b^
1	146.154	55.084	1.432	131.65	0.836	HBAL, HY, HY, HY, RA
2	146.512	54.726	1.422	131.23	0.833	HBAL, HY, HY, HY, RA
3	147.116	54.122	1.453	132.2	0.827	HBD, HY, HY, HY, RA
4	147.381	52.857	1.453	132.58	0.827	HBAL, HY, HY, HY, RA
5	148.361	52.877	1.452	132.54	0.828	HBAL, HY, HY, HY, RA
6	158.591	42.647	1.694	143.98	0.756	HBD, HY, HY, HY, RA
7	159.501	41.737	1.712	144.88	0.75	HBD, HY, HY, HY, RA

^a^ Null cost = 201.238, fixed cost = 115.403, configuration cost = 13.075. All units are in bits. ^b^ HBAL, hydrogen-bond acceptor lipid; HY, hydrophobic feature; RA, ring aromatic.

**Table 3:** Docking scores of reference compounds and the final hits obtained after the docking study.

Structure	Chemical Name	Fit Value	GOLD Score	MolDock Score	Re-Rank Score
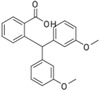	(NSC-21270 or CID 228386)	9.059	59.82	−109.786	−81.861
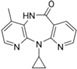	Nevirapine	6.22	54.34	−80.422	−69.192
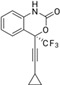	Efavirenz	7.35	60.34	−90.264	−63.537
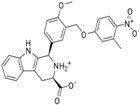	ZINC09033850	7.855	50.50	−132.503	−78.049

**Table 4:** The binding scores of identified leads from PBVS.

Ligand	MolDock Score	Re-Rank Score	Docking Score	Similarity Score
ZINC00118327	−94.3105	−68.3936	−537.27	−442.8
ZINC02145802	−135.01	−46.6255	−523.953	−388.385
ZINC02146330	−148.393	−105.048	−572.967	−428.346
ZINC03882136	−106.935	−89.9424	−505.056	−397.593
ZINC03999710	−99.4136	−89.1023	−468.8	−365.662
ZINC04042111	−103.784	−86.9628	−558.144	−456.27
ZINC04042113	−97.2975	−85.2139	−492.057	−434.711
ZINC04042592	−133.52	−115.917	−530.436	−393.823
ZINC04042644	−110.695	−83.6879	−540.142	−430.804
ZINC04044269	−102.001	−88.9174	−529.073	−428.531
ZINC08590027	−85.4343	−76.061	−499.522	−410.26
ZINC08606304	−117.548	−85.374	−599.476	−478.438
ZINC08964648	−84.2768	−88.907	−582.527	−501.149
ZINC08964651	−103.4529	−104.479	−584.946	−493.032
ZINC08964652	−96.8387	−90.599	−545.242	−452.769
ZINC08964664	−102.029	−129.786	−566.44	−468.224
ZINC09033565	−97.1431	−85.9664	−435.762	−329.149
ZINC09033687	−82.9655	−77.765	−524.96	−440.625
ZINC09514115	−109.965	−90.9183	−569.119	−456.021
ZINC12661581	−101.547	−127.691	−692.064	−579.885
ZINC12661651	−66.9044	−119.657	−548.942	−483.748
ZINC12661660	−130.781	−95.7949	−573.955	−445.823
ZINC12661665	−125.599	−94.9555	−569.362	−446.274
ZINC12661671	−96.574	−85.7076	−568.056	−475.063
ZINC12661673	−94.3644	−81.02	−499.607	−401.587

## Data Availability

The data presented in this study are available in supplementary material.

## Supplementary Materials

Figure S1. The ligand pharmacophore mapping of the external test set of compounds on the pharmacophore model. Table S1. Scores and data related to training set. Table S2. Test set prediction on training set pharmacophore. Table S3. Mapping of standard compounds and estimated activities using hypothesis 1.

## Sample Availability

Samples of the compounds are not available in this manuscript from the authors.
