# Supplementary material for: Identification of 3-((1-(Benzyl(2-hydroxy-2-phenylethyl)amino)-1-oxo-3-phenylpropan-2-yl)carbamoyl)pyrazine-2-carboxylic Acid as a Potential Inhibitor of Non-Nucleosidase Reverse Transcriptase Inhibitors through InSilico Ligand- and Structure-Based Approaches
Source: Molecules. 2021 Aug 30;26(17):5262. doi: 10.3390/molecules26175262 (PMC8433663; doi:10.3390/molecules26175262)
Supplement: Supplementary file 1 [file molecules-26-05262-s001.zip › molecules-1348964-supplementary.pdf]

Pharmacophore modeling, docking and virtual screening approaches in the discovery of novel Non-Nucleoside Reverse Transcriptase Inhibitors (NNRTI's)

Supporting Information Tables

Table S1. Scores and data related to training set.

Name	IC_{50} (mM) Reported[a]	IC_{50} (mM) Estimated[b]	Fit Value[c]	Error	Activity Scale Est.[d]	Observed Scale Rep.[d]	HBA lipid[e]	RA[e]	HY[e]
**1**	0.008	0.051	10.73	-6.4	++	+++	1	1	101
**7**	1.585	0.16	10.22	-9.7	++	++	1	1	101
**8**	3.98	2.3	9.07	-1.7	++	++	1	1	101
**20**	0.159	0.39	9.85	2.5	++	++	0	1	101
**26**	0.398	0.084	10.51	-4.7	+++	++	1	0	101
**27**	0.0004	0.0009	13.45	2.4	++	+++	1	1	111
**40**	1.995	0.18	10.18	-11	++	++	1	1	101
**42**	1	0.38	9.85	-2.6	++	++	1	0	101
**43**	1.995	0.13	10.31	-15	++	++	1	1	111
**46**	0.398	0.37	9.87	-1.1	++	++	1	1	111
**54**	0.05	0.2	10.14	4	++	++	1	1	101
**57**	0.003	0.03	10.96	10	+++	+++	1	0	111
**60**	0.004	0.0035	11.89	-1.1	+++	+++	1	1	111
**61**	0.001	0.0018	12.18	-5.5	+++	+++	1	1	111
**63**	0.005	0.003	11.97	-1.7	++	+++	1	1	101
**66**	0.05	0.012	11.43	-4.9	++	++	1	1	111
**67**	1.585	0.3	9.96	-5.3	++	++	1	1	111
**68**	1.258	1	9.43	-1.2	++	++	1	1	111
**76**	0.199	0.16	10.24	-1.3	+	++	1	1	111

**83**	100	36	6.97	-2.8	+	+	1	1	101
**86**	5.012	2.1	9.12	-2.4	+++	++	1	1	111
**88**	0.003	0.01	10.42	3.2	++	+++	1	1	111
**92**	0.01	0.2	10.15	2.1	+++	+++	1	0	111
**94**	0.02	0.13	10.34	6.3	++	+++	1	1	111
**95**	0.316	0.21	10.12	-1.5	++	++	1	0	111
**96**	0.079	0.17	10.22	2.1	++	++	1	1	111
**97**	0.1	0.16	10.22	1.6	++	++	1	1	111
**99**	0.02	0.041	10.82	2	+++	++	1	1	111
**102**	0.158	0.12	10.35	-1.3	+++	++	1	1	101
**103**	0.032	0.14	10.28	4.5		++	1	1	111

[a] Reported activity, [b] Estimated Activity, [c] Ligand pharmacophore mapping based fit value, [d]Activity Scale HA (highly active, +++, 0.0004-0.01), MA (moderately active, ++, 0.01-5) and LA (least active, +, 5-100) according to their reported and estimated biological activity. [e] Feature Mapping based on Ligand pharmacophore mapping HBA, RA, HY.

Table S2. Test set prediction on training set pharmacophore.

Name	IC_{50} (nM) Reported[a]	IC_{50} (nM) Estimated[b]	Fit Value[c]	Activity Scale[d]	Observed Scale[d]	Mapped Features[e]		
				cat[i]	obs[ii]	HBA	RA	HY
**2**	0.004	0.031	6.866	++	+++	1	1	111
**3**	0.025	0.0099	7.146	+++	++	1	1	111
**4**	0.002	0.0005	8.415	+++	+++	1	1	111
**5**	0.316	0.04	8.851	+++	++	1	1	111
**6**	0.01	0.0004	8.561	+++	+++	1	1	111
**9**	0.006	0.0098	7.149	+++	+++	1	1	111
**10**	0.006	0.0051	7.429	+++	+++	1	1	111
**11**	0.002	0.0168	6.914	++	+++	1	1	111
**12**	0.005	0.0008	8.257	+++	+++	1	1	111
**13**	0.004	0.001	8.16	+++	+++	1	1	111
**14**	0.199	0.0002	8.76	+++	++	1	1	111
**15**	1.585	0.5	8.752	++	++	1	1	111
**16**	0.004	0.003	7.662	+++	+++	1	1	111
**17**	0.013	0.0066	7.323	+++	++	1	1	111
**18**	1.259	0.305	8.681	+++	++	1	1	111
**19**	0.1	0.0005	8.464	+++	++	1	1	111
**21**	0.04	0.0002	8.816	+++	++	1	1	111
**24**	0.013	0.0004	8.592	+++	++	1	1	111
**25**	0.008	0.0005	8.426	+++	+++	1	1	111
**28**	0.002	0.0005	8.468	+++	+++	1	1	111
**29**	0.002	0.001	8.162	+++	+++	1	1	111

**30**	0.002	0.0103	7.126	+++	+++	1	1	111
**32**	0.002	0.09	6.467	++	+++	1	1	111
**31**	0.002	0.0043	7.51	+++	+++	1	1	111
**33**	0.004	0.0004	8.516	+++	+++	1	1	111
**34**	0.002	0.0006	8.352	+++	+++	1	1	111
**35**	0.05	0.0495	6.446	++	++	1	1	111
**36**	0.003	0.0006	8.367	+++	+++	1	1	111
**37**	0.079	0.0005	8.41	+++	++	1	1	111
**38**	0.398	0.088	8.519	+++	++	1	1	111
**39**	0.012	0.0084	7.215	+++	++	1	1	111
**41**	0.126	0.0044	7.496	+++	++	1	1	111
**46**	0.398	0.025	8.593	+++	++	1	1	111
**47**	0.398	0.176	7.579	+++	++	1	1	111
**53**	0.008	0.0035	7.599	+++	+++	1	1	111
**56**	0.001	0.0021	7.809	+++	+++	1	1	111
**49**	0.063	0.0377	6.4095	++	++	1	1	101
**54**	0.05	0.0007	8.3	+++	++	1	1	111
**44**	0.04	0.1	6.932	++	++	1	1	101
**50**	0.251	0.247	6.7159	++	++	1	1	111
**55**	0.01	0.0033	7.625	+++	+++	1	1	111
**58**	0.039	0.0019	7.861	+++	++	1	1	111
**45**	0.079	0.045	6.4	++	++	1	1	101
**51**	0.006	0.06	6.7159	+++	++	1	1	111
**52**	0.158	0.0055	8.3653	+++	++	1	1	111
**62**	0.003	0.0003	8.719	+++	+++	1	1	111
**64**	0.002	0.0028	7.687	+++	+++	1	1	111
**65**	0.005	0.0015	7.956	+++	+++	1	1	111

**67**	1.585	0.251	5.449	++	++	1	1	111
**71**	0.079	0.0044	7.498	+++	++	1	1	111
**72**	0.079	0.0014	7.991	+++	++	1	1	111
**73**	0.001	0.0009	8.175	+++	+++	1	1	111
**74**	0.012	0.0042	7.519	+++	++	1	1	111
**75**	0.158	0.0007	8.267	+++	++	1	1	111
**76**	1	0.239	7.956	+++	++	1	1	111
**77**	0.199	0.022	7.651	+++	++	1	1	111
**78**	0.063	0.0034	7.608	+++	++	1	1	111
**79**	0.001	0.0011	8.113	+++	+++	1	1	111
**80**	0.006	0.0012	8.069	+++	+++	1	1	111
**81**	0.003	0.0015	7.956	+++	+++	1	1	111
**82**	0.008	0.0012	8.069	+++	+++	1	1	111
**84**	0.063	0.049	6.829	++	++	1	1	111
**85**	0.039	0.0005	8.48	+++	++	1	1	111
**86**	0.015	0.015	7.435	++	++	1	1	111
**88**	0.016	0.0353	6.592	++	++	1	1	111
**90**	0.01	0.0003	8.626	+++	+++	1	1	111
**91**	0.003	0.0006	8.329	+++	+++	1	1	111
**92**	0.061	0.003	7.669	+++	++	1	1	111
**94**	0.199	0.0022	7.792	+++	++	1	1	111
**99**	0.063	0.003	7.662	+++	++	1	1	111
**101**	0.079	0.0005	8.486	+++	++	1	1	111

[a] Reported activity, [b] Estimated Activity, [c] Ligand pharmacophore mapping based fit value, [d]Activity Scale HA (highly active, +++, 0.0004-0.01), MA (moderately active, ++, 0.01-5) and LA (least active, +, 5-100) according to their reported biological activity ([i] Estimated scale by catalyst software, [ii]Reported scale). [e]Feature Mapping based on Ligand pharmacophore mapping.

Table S3. Mapping of standard compounds and estimated activities using hypothesis 1.

**Comp.**	**Fit Value**	**Estimated IC_{50} in (µM)**	**Reported IC_{50} in (µM)**
Delavirdine	7.34	0.058	0.0161
DPC083	7.13	0.062	0.0004
HETP-1	6.06	1.117	5.06
Nevirapine	6.22	0.759	0.18
Efavirenz	7.35	0.057	0.004
TMC125	6.34	0.515	0.0014
L697.661	7.90	0.016	0.23
GW8248	7.47	0.043	0.0046

Figure S1. The ligand pharmacophore mapping of the external test set of compounds on the pharmacophore model.

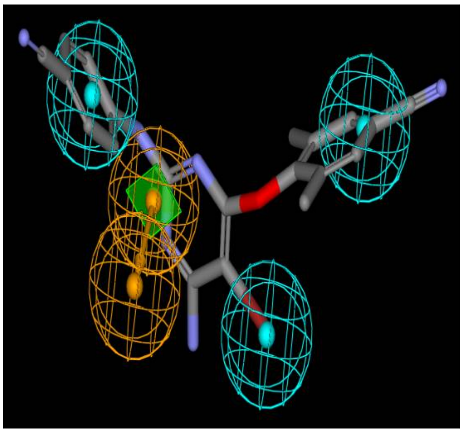

a. Mapping of TMC-125 on Hypo-1.

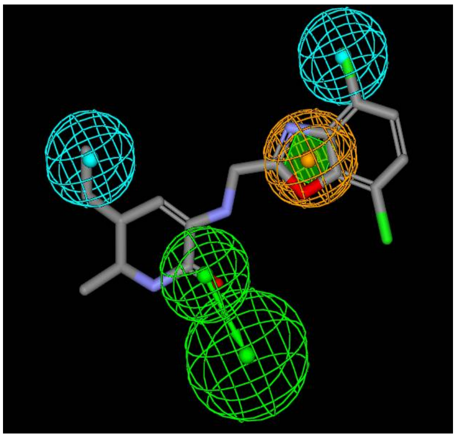

b. Mapping of L697,661 on Hypo-1.

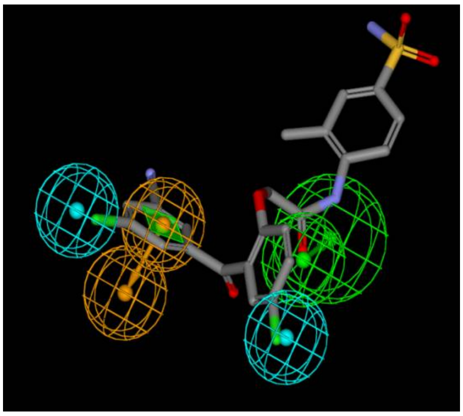

c. Mapping of GW8248 on Hypo- 1.

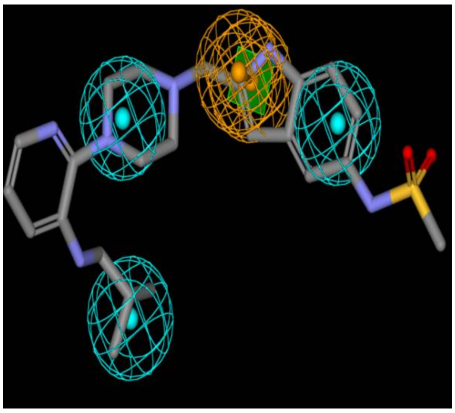

d. Mapping of Delavirdine on Hypo- 1.

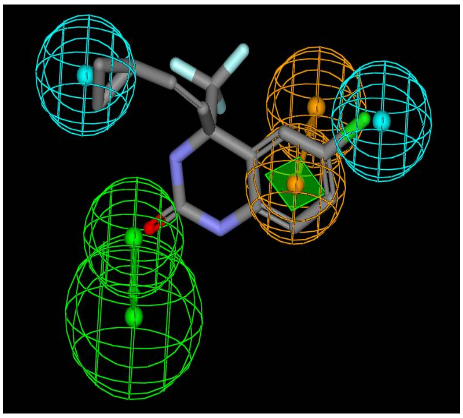

e. Mapping of DPC083 on Hypo- 1.

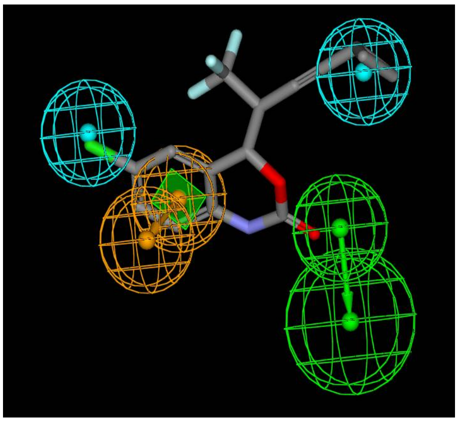

f. Mapping of Efavirenz on Hypo- 1.
